# Reducing Sodium in Foods: The Effect on Flavor 

**DOI:** 10.3390/nu3060694

**Published:** 2011-06-20

**Authors:** Djin Gie Liem, Fatemeh Miremadi, Russell S. J. Keast

**Affiliations:** School of Exercise and Nutrition Sciences, Centre for Physical Activity and Nutrition Research, Sensory Science Group, Deakin University, Melbourne, 3125, VIC, Australia; Email: gie.liem@deakin.edu.au (D.G.L.); fmir@deakin.edu.au (F.M.)

**Keywords:** salt taste, flavor, sensory

## Abstract

Sodium is an essential micronutrient and, via salt taste, appetitive. High consumption of sodium is, however, related to negative health effects such as hypertension, cardiovascular diseases and stroke. In industrialized countries, about 75% of sodium in the diet comes from manufactured foods and foods eaten away from home. Reducing sodium in processed foods will be, however, challenging due to sodium’s specific functionality in terms of flavor and associated palatability of foods (*i.e.*, increase of saltiness, reduction of bitterness, enhancement of sweetness and other congruent flavors). The current review discusses the sensory role of sodium in food, determinants of salt taste perception and a variety of strategies, such as sodium replacers (*i.e.*, potassium salts) and gradual reduction of sodium, to decrease sodium in processed foods while maintaining palatability.

## 1. Introduction

Sodium chloride (NaCl) is the prototypical stimulus for salty taste [1]. Sodium improves the sensory properties of foods, by increasing saltiness, decreasing bitterness, and increasing sweetness and other congruent flavor effects [2]. Factors which determine an individual’s liking and acceptance of salty foods are poorly understood, however it is thought that environmental factors such as the level of sodium in foods and habitual diet play a significant role [3,4]. While sodium is essential for normal human functioning, current sodium intakes far exceed recommendations for good health [5]. Excessive sodium intake is associated with an increase in blood pressure, which is a major cause of cardiovascular diseases. It has been estimated that 62% of stroke and 49% of coronary heart disease is caused by high blood pressure [6]. Excess sodium consumption has also been associated with numerous other negative health effects, including gastric cancer [7], decreased bone mineral density [8] and possibly obesity [9].

A report by Asaria *et al.* [10] calculated that a modest 15% reduction in population sodium intake could prevent 8.5 million cardiovascular-related deaths worldwide over 10 years. A meta analysis prepared by the WHO concludes that there is strong evidence for the cost effectiveness of national sodium reduction strategies [10,11]. For example, cardiovascular diseases are the most expensive health issue accounting for 11% of total health expenditure around the world [12]. The average sodium reduction strategy is expected to cost only 0.3% of the current expenditure on hypertension control program in conjunction with other cardiovascular-associated costs worldwide [10]. Lowering sodium intake is beneficial for hypertensive and normotensive people although it affects hypertensive people to a greater degree. 

Despite the negative health consequences and associated health care costs of high sodium consumption, humans consume well above the recommended levels in most developed nations, making sodium reduction a priority for public health [11,13]. For this reason, a range of strategies to reduce sodium in different foods have been applied. However, success is often limited as sodium reduction has adverse affects on taste quality and flavor perception [14]. The objective of this paper is to (1) review the role sodium plays in the flavor of food, (2) to assess strategies to reduce sodium in processed foods.

## 2. Physiological Role of Sodium in the Body, Recommended Sodium Intake, and Sodium Consumption Trends

Within the body, sodium regulates extracellular volume, maintains acid-base balance, neural transmission, renal function, cardiac output and myocytic contraction [1]. World Health Organization (WHO) recommendations indicate that, in order to prevent chronic diseases, an adult upper daily limit intake of sodium should be less than 87 mmol Na/day (<5 g NaCl/day) [15]. The average US sodium intake is estimated to be 140–160 mmol Na/day (8.2–9.4 g NaCl/day) [13], United Kingdom 161 mmol Na/day (9.4 g NaCl/day) [16] and Asian countries higher than 206 mmol Na/day (12.0 g NaCl/day) [15]. These studies illustrate consumption levels well in excess of sodium required for optimum health. In westernized countries, approximately 75% of sodium in the diet comes from processed foods, and foods eaten away from home [15,17]. The processing of foods often involves adding sodium to foods for a variety of flavor or processing reasons. For example chick peas, sweet corn and peas, which have naturally very low sodium content, have 10 to 100 times more sodium post-processing (Table 1) [15].

It is believed that the relatively high sodium intake of almost all societies today became common beginning between 5000–10,000 years ago [18,19]. It is generally thought that food preservation was the main driver of high sodium consumption in the early days [20]. This early use of sodium is likely to be the origin of the current high consumption of sodium. Interestingly, sodium consumption did not change dramatically over time. For example, in 300 B.C. the average daily sodium intake in certain parts of China was approximately 3000 mg Na/day (7.6 g NaCl/day) for women and 5000 mg Na/day (12.7 g NaCl/day) for men [21]. In his book “Neptune’s gift: a history of common salt”, Multhauf estimated that the average salt consumption in 1850 in Britain and France was about 4000–5000 mg Na/day (10.2–12.7 g NaCl/day) [20], which is rather similar to the current intake of sodium [22]. In 2010, Bernstein and Willett [23] analyzed 38 studies, published between 1957 and 2003, which investigated sodium consumption, and found that individuals consistently consumed about 3700 mg Na/day (9.4 g NaCl/day) throughout this period. Despite the absence of any great increase in sodium consumption in the past centuries, salt intake has remained too high as people transitioned from preserved foods to modern processed foods [23].

**Table 1:** Comparison of the sodium content of some of the “natural” and processed foods [15].

Food item	Description	Sodium content (mg/100 g)
Beef	Topside, roast, lean and fat	48
	Corned beef, canned	950
Bran	Bran, wheat	28
	Bran Flakes	1000
Cheese	Hard, average	620
	Processed	1320
Chick-peas	Dried, boiled in unsalted water	5
	Canned, re-heated, drained	220
Potato	Raw, boiled in unsalted water	9
	Canned, re-heated, drained	250
Peas	Raw, boiled in unsalted water	Trace
	Canned, re-heated, drained	250
Potato chips	Homemade, fried in blended oil	12
	Oven chips, frozen, baked	53
Salmon	Raw, steamed	110
	Canned	570
	Smoked	1880
Sweet corn	On-the-cob, whole, boiled in unsalted water	1
	Kernels, canned, re-heated, drained	270
Tuna	Raw	47
	Canned in oil, drained	290
	Canned in brine, drained	320

**Figure 1:**
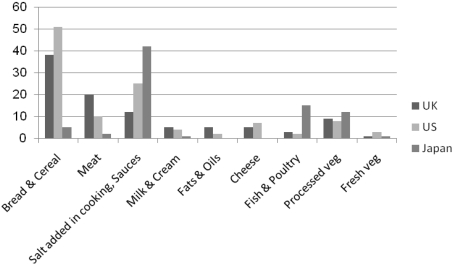
Percentage contribution of food types to average daily intake of sodium [15,24].

Cereals and cereal products such as bread, breakfast cereals, biscuits and cakes, contribute about 30–50% of the estimated total intake of sodium in UK and US (see Figure 1). In Asian countries, such as Japan, a large proportion of dietary sodium comes from sodium added in cooking (*i.e.*, soy sauce) [15,24]. Fast foods also contribute a significant amount to the daily sodium intake, e.g., one large slice of pizza alone contributes 1000 mg sodium or 43% of upper daily limit intake of sodium (2300 mg Na/day; 5.8 g NaCl/day) [25]. 

## 3. Flavor Perception

Flavor is a unitary percept, however what we perceive as an unitary whole is a combination of inputs from independent sensory systems: taste, smell and chemical irritation (part of the sense of touch) [14]. For example, when we consume an orange flavored soft-drink, the sense of taste is activated by non-volatile sugars and acids, the sense of smell by volatile aromas added to characterize the soft-drink (orange), and the chemical irritation by carbon dioxide. We do not perceive sweetness independently of orange aroma or carbon dioxide tingle; we perceive the sensations simultaneously as an orange flavored soft-drink. This central integration of taste, smell and chemical irritation ensures that there is ample opportunity for interactions between the senses. Removing or reducing one component of flavor, for example sweet taste can have effects beyond simply loss of sweetness; it can influence the entire flavor profile [2].

## 4. Taste Perception

The sense of taste presumably evolved to detect both toxins and nutrients in foods. To perform this task the taste system is subserved by five taste qualities: sweet, elicited by sugars indicating carbohydrates in foods; umami, elicited by glutamic acid and other amino acids indicating protein in foods; sour, elicited by protons indicating acidic foods; bitter indicating toxic foods; and salt, elicited by sodium content of foods [26].

Taste receptor cells, which mediate the sense of taste, are located throughout the oral cavity. Most taste receptor cells are components of taste buds, which are clustered on three types of papillae (*i.e.*, fungiform, foliate, and circumvallate) located on the tongue [27]. Papillae contain several hundred taste buds, each of which is composed of 50 to 150 taste receptor cells. The taste receptors at the apical end of the taste receptor cells are exposed to the internal environment in the oral cavity. When food or drink enters the mouth, chemicals from those foods may activate taste receptors. The chemical signal is converted to an electrical signal and sent via the seventh, ninth and tenth cranial afferent nerve fibers to the gustatory processing regions of the brain [2,26].

Perceived taste is characterized by four separate attributes—quality, intensity, temporal and spatial patterns [2]. Taste quality is the most important defining feature of taste sensation and is defined as a descriptive noun given to categorize sensations that taste compounds elicit: sweet, sour, salty, bitter and umami. Perceived intensity is related to the strength of the taste sensation and, when plotted against tasted concentrations, creates a psychophysical function. Temporal pattern is used to describe the time course of the taste perception [2,14], and spatial topography relates to location and localizability of taste sensation. If we use sodium as an example, the taste quality elicited is saltiness, the intensity of saltiness varies with the concentration of sodium in a food, and the time course of saltiness varies from when you put the salty food in your mouth through to mastication, swallowing and aftertaste. The location of saltiness originates throughout the entire oral cavity where taste receptor cells are located. It is important to note that there is no such thing as a tongue map, saltiness can be perceived across the tongue, rather than by specific areas on the tongue as wrongly suggested by the tongue map [27].

### Salt Taste Perception

The evolutionary importance of sodium is illustrated in the fact that one taste quality is devoted to identifying sodium in foods. Lithium also has a pure salt taste, but due to toxicity will not be an approved food ingredient, however other minerals such as potassium may also have a salty component to their taste [28].

The exclusivity of sodium as a stimulus for salty taste can be explained by its unique sodium-specific transduction mechanism involving epithelial sodium channels (ENaCs) on the taste receptor cells [29]. There are two ENaCs subtypes, one specific for sodium, which is activated at low sodium concentrations and is believed responsible for the appetitive nature of salt taste. The second ENaCs is permeable to multiple cations, activated at higher sodium concentrations, and is believed responsible for the aversive nature of cations. The seminal research revealing putative salt taste mechanisms was based on a mouse model, but there is little reason to believe the mechanisms would differ in humans [2,26].

**Figure 2:**
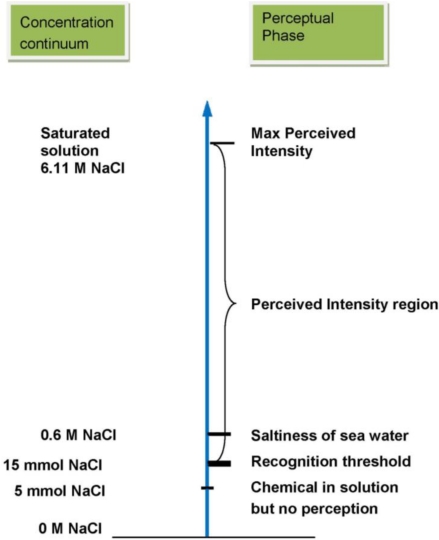
Scale diagram of taste perception as concentration increases (adopted from [26]).

Salt taste perception begins when sodium activates ENaCs on taste receptors and an afferent signal is sent to gustatory processing regions of the brain. At low sodium concentrations, the afferent signal may be too weak and not able to produce a noticeable difference from a similar solution without sodium. As the concentration of sodium increases the afferent signal strength will increase and reach a level where an individual will be able to discriminate a sodium solution from water, but remain unable to identify the taste quality. This is known as the detection threshold and is often used as a measure of individual sensitivity to sodium. Actual identification of saltiness occurs when the concentration of sodium is high enough not only to activate the taste receptors, but also produce electrical impulses, which can be carried via sensory neurons to the brain where they are decoded and after which the taste quality can be identified. This is known as the recognition threshold. The sodium concentrations above the recognition threshold are in the range of perceived saltiness, which is termed suprathreshold (Figure 2) [2,26]. The concentration of sodium required to elicit saltiness will vary considerably between food matrices, e.g., it is easier to detect 50 mM NaCl in an aqueous solution than in a bread matrix.

## 5. Taste Interactions

The perceived intensity of a single compound in an aqueous solution increases as its concentration increases. However, in everyday life we mostly consume mixtures, rather than singular taste stimuli, notable exceptions being sucrose (table sugar) and NaCl (sodium chloride or table salt). The perceived intensity of mixtures may be additive or non-additive, which result in suppression or enhancement outcomes. Suppression takes place when the total intensity of a mixture, existing of two or more suprathreshold stimuli of the same modality, is less than the sum its components (e.g., 1 + 1 < 2) [14]. Enhancement is the opposite of suppression (the intensity of a mixture is greater than the sum of its components [14].

### 5.1. Levels of Taste Interactions

Interactions between taste compounds such as mixture suppression and enhancement can occur at three different levels including chemical interactions, oral physiological interactions and central cognitive interactions. Chemical interactions may occur in the food matrix, for example gluten in bread may bind sodium, making it unavailable for taste reception [14].

Oral physiological interactions involve one compound interfering with taste receptor cells or taste transduction mechanisms associated with a second compound. These are classified as peripheral interactions, as they occur at the epithelial/cellular level. An example of oral peripheral interaction is the effect sodium salts have on bitterness, where sodium interferes with the bitter taste transduction prior to the taste signal being sent to processing regions of the brain [2,30].

To demonstrate this peripheral effect, Kroeze and Bartoshuk [31] used a split-tongue technique in which sodium salt and a bitter stimulus were simultaneously applied to separate parts of the tongue, or were applied as a mixture to the same part of the tongue. The intensity of bitterness was reduced more when the stimuli were applied to the tongue in a mixture together, compared to when salt and bitter taste stimuli were applied to separate parts of the tongue. This illustrated that sodium interferes with the bitter taste transduction, although the mode of action in the periphery is unknown.

Cognitive interactions refer to central processing of taste stimuli after afferent signals are sent to taste processing regions of the brain. Mixture suppression, which takes place after stimuli were applied to separate parts of the tongue, is an example of cognitive interaction [31,32].

### 5.2. Taste Interactions Involving Saltiness

**Figure 3:**
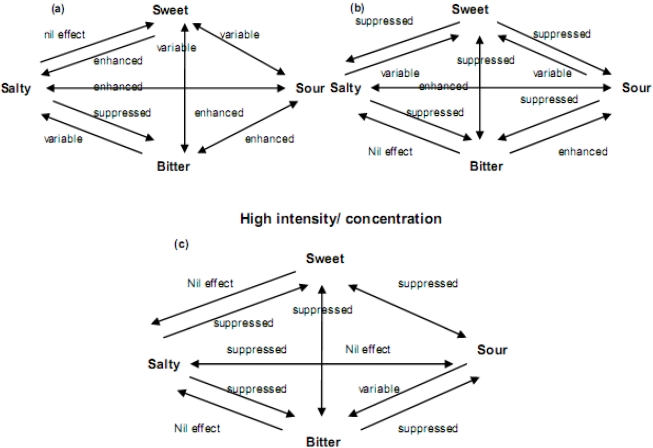
Schematic review of binary interactions of taste qualities at different levels of intensity/concentration, Adapted with permission from Elsevier [2].

When two compounds with different taste qualities are mixed, a number of interactions may occur, including non-monotonic (both enhancement and suppression) and asymmetrical intensity shifts [2,30]. In food matrices, sodium salts may influence other taste qualities independent of intensity/concentration. For example, it has been suggested that saltiness is suppressed at high concentrations of of NaCl and KCL, but enhanced at low concentrations of NaCl and KCl saltiness [2]. Salt and sour taste mixtures symmetrically affect each other’s intensity with enhancement at low intensities/concentrations and suppression or no effect at high intensities/concentrations [33,34]. Bitterness is suppressed by sodium at all intensities/concentrations, while salt taste is less affected by bitterness. Sodium enhances sweetness at low intensities/concentrations, has variable effects through the moderate intensity/concentration range, and is suppressive or has no effect on sweetness at higher intensity/concentration. Sweetness suppresses salty taste at moderate intensities [2]. Figure 3 shows a schematic overview of binary interactions of taste qualities at different levels of concentrations. These schematic reviews are just indications of what happens to taste qualities when two are mixed. Variations, depending on the food matrix, may occur.

Interactions between tastes get more complex when three taste qualities interact, or when more complex food matrices are involved [32]. Breslin *et al.* [35] investigated the interaction between sodium (salt), sucrose (sweet) and urea (bitter) in a mixture. They found that perceived bitterness is suppressed when sodium is added to a bitter-sweet mixture. Due to the decreased perceived bitterness, perceived sweetness increased. The latter was a result of the bitterness being less able to reduce perceived sweetness. This interaction takes place at a cognitive level. These findings are in line with Gillette *et al.* [36], who suggested that addition of NaCl to three soups decreased bitterness and increased sweetness. Similarly, Fuke and Konosu [37] reported that addition of umami tasting sodium salts of 5′-ribonucleotides reduced bitterness and increased sweetness in an artificial prawn extract. Pangborn, who has been recognized as one of the most influential researchers in the area of taste interaction [38,39,40], performed a series of experiments in the early 1960s investigating sucrose, citric acid and NaCl taste interrelationships. Several different food matrixes were used, e.g., pear nectar [41] tomato juice [42] and lima bean puree [43]. The results from the food matrix mirrored those in simple aqueous media [38,39,44], and from other studies [35,36].

**Figure 4:**
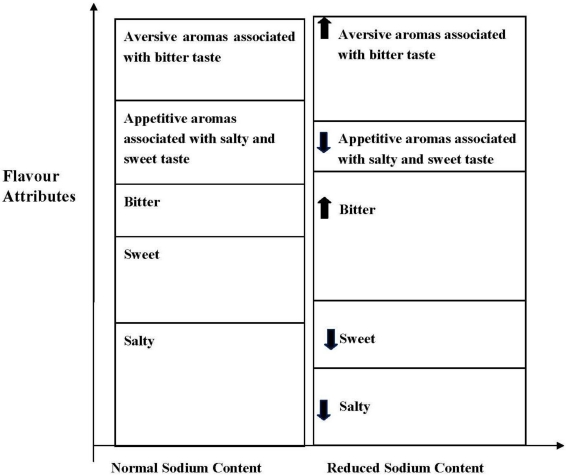
Influence of sodium reduction on flavor. The height of the boxes making up total flavor reflects intensity of the named attribute in standard or reduced sodium food [14,35].

Overall, reduction of sodium from food will have multiple flavor effects, as shown in Figure 4. The primary effect will be loss of saltiness. There may also be an increase of bitterness, due to the effect of sodium as an effective bitterness inhibitor; removing sodium will cause bitterness to be released from suppression. An increase in bitterness will result in mixture suppression, thereby decreasing sweetness [35]. In terms of taste liking, reducing sodium may decrease appetitive salt and sweet taste, and increase aversive bitter taste. A reduction in appetitive salty and sweet taste, may also result in a decreased perception of appetitive aromas associated with those tastes [14,35]. Overall, reducing sodium in foods not only reduces perceived saltiness, but is also associated with a wide range of complex taste interactions, which may negatively impact the liking of foods. Without sufficient knowledge about taste interactions the search for sodium replacers and other strategies to decrease sodium in industry sourced foods will be challenging.

## 6. Factors Affecting Sensitivity and Preference for Salt Taste in Foods

Supermarket shelves contain very few low sodium processed foods, which makes it difficult for consumers to follow a low sodium diet [45]. Individuals continue to consume large amounts of sodium and liking is preferentially directed to sodium-rich foods despite knowledge of the negative health effects [6,46,47]. Little is known about the factors which affect an individual’s salt taste sensitivity, liking and selection of salty foods, and whether there is an association between oral sodium sensitivity and liking of salty foods.

### 6.1. Genetic Factors

To our knowledge only two studies have tested the association between heritability and salt taste sensitivity as assessed by recognition threshold [48,49]. They concluded that salt taste sensitivity had no major heritable components and environmental factors may play a greater role in salt taste sensitivity. No measures of salt preference were included in these studies.

### 6.2. Environmental Factors

Environmental factors appear to have a larger influence on salt taste preference, than genetic factors [3]. Infants and children’s liking for salt in specific foods is thought to be influenced by consumption of salty foods commonly available after 6-months of age [4], when infants are able to detect and preferentially ingest NaCl solutions compared to water. This developmental change is thought to be a result of experience, and salt taste preference is said to be “learned” rather than innate [4]. Further evidence of the role of environmental factors in salt taste preferences are based on observations that exposure to sodium determines salt taste preferences [50]. Subjects on a sodium reduced diet experienced increased saltiness in foods and a decreased liking for higher concentrations of sodium chloride in foods [51,52]. Some researchers suggest a link between sodium intake and both salt taste sensitivity and preference [51,52], which is likely caused by the sensory perception of salt [53]. It is also believed that changes in salt taste preference were due to sensory experience of salt taste rather than actual amount of sodium consumed.

The evidence, however, is not conclusive, and other research has shown no link between salt taste sensitivity and consumption or liking of salty food [54], or between consumption of reduced sodium diet and increasing preference for salty foods [55,56]. However, these studies have generally involved a dramatic sodium reduction, e.g., a 50% reduction over a short period of time. Bertino *et al.* [51] hypothesized a biphasic response to sodium reduction which is characterized by an initial phase of increased attraction to salt taste followed by a decreased preference for salt which would explain the discrepancies in results.

## 7. Methods of Sodium Reduction

### 7.1. Sodium Restricted Diet

A simplistic view on sodium reduction is to just tell consumers they eat too much sodium and expect them to change their eating behavior. Community-based intervention trials, however, have demonstrated that only 20–40% of participants were able to reduce their sodium intake below the recommended upper limit of 100 mmol Na/day (5.8 mg NaCl/day) despite intense counseling [45]. Due to the need for counseling, this intervention is not feasible at a population level. 

Sodium reduced diets are difficult to maintain as they often require a change in dietary behavior for example, actively choosing low salt foods of which there is very limited choice on supermarket shelves [57]. It is widely accepted that sodium reduction could be more effectively achieved by reducing the sodium content of processed foods and by applying multisensory principles, e.g., enhancing aromas, to optimize the flavor characteristics of the foods, rather than by just giving dietary advice alone [1,57,58].

### 7.2. The United Kingdom Strategy—Sodium Reduction by Stealth

The UK approach is based around stealth reduction methods which refers to a gradual reduction of salt in processed foods that is unnoticeable to consumers [59]. Grigis *et al.* [60] gradually reduced the sodium content of white bread by 25% over a period of 6 weeks and consumers generally could not notice a difference in flavor. For food industry this would mean that they can meet sodium reduction targets by gradually decreasing sodium in their product over the course of a couple of years, without losing consumers. This strategy has been successful with the sodium content of many processed foods in supermarkets being reduced by 20–30% in the past 3 years. These results are expected to be duplicated when revised targets are set for a further 10–20% reduction to meet the UK’s daily intake target of 6 g/day by 2012 [61]. The reduction by stealth approach has the benefit of not requiring any behavior change from consumers, which is traditionally difficult to achieve. This approach has resulted in the UK population intake of NaCl reducing by approximately 1 g/day [24,57].

### 7.3. Use of Salt Substitute

An ideal situation would be the replacement of sodium with a compound that elicits a similar pure saltiness when consumed. However, such replacements appear improbable due to the specificity of sodium to the ENaC responsible for salt taste. Sodium chloride replacers such as potassium chloride, calcium chloride and magnesium sulfate have been used to replace or enhance salt taste in a number of food products [28]. While these compounds do contribute a certain salty taste quality, they may also provide undesirable after tastes such as bitter, metallic and astringent tastes, which has limited their current use in food manufacturing [62,63]. These other non-sodium cations are believed to activate a second type of salt taste ENaC, which is non-specific and believed responsible for the off-tastes and flavors. So while the use of other cations is appealing, in particular potassium due to the added health benefit of increasing dietary potassium, the concentration which can be used in processed food will be limited [63]. Potentially, the intensity of perceivable bitter taste and associated off flavors can be decreased by “bitter blockers”, or sweeteners such as sucrose and thaumatin, which is an intensely sweet tasting protein [64].

Furthermore, food grade acids have been shown effective in enhancing saltiness of sodium. Little and Brinner [65] observed the effects of modifying NaCl and citric acid content of tomato soup on both taste preference and saltiness [65]. Saltiness intensity increased with increasing citric acid concentration. Taste preferences followed a hyperbolic function with an initial increase to a point of maximum taste preference (between 0.15–0.3% citric acid) and then dropping quite rapidly thereafter. Maximum taste preference was reached at both moderate NaCl (0.6%) and acid (0.3%) concentrations. It is also interesting to note that a low NaCl soup with citric acid could match or exceed the liking of a high NaCl soup [65]. A similar study investigated the effects of taste mixtures of NaCl and a combination of acetic and lactic acid in both simple solutions and bread [66]. As in the soup study, increasing concentrations of acid caused increased perceived saltiness at all salt variations in bread samples. Highest pleasantness rating was reached at a moderate acid (0.6%) and highest NaCl (1.2%) concentration in bread. The maximum taste preference of both studies involved only moderate acid concentrations. This suggests the use of acids to enhance salty taste is limited to lower concentrations, because sour notes may dominate and affect hedonic responses at higher concentrations. However, there may be some food matrices in which the use of acids would not be successful. 

### 7.4. Other Approaches to Sodium Reduction

Due to the influence of sodium reduction on the total sensory profile of foods, the addition of flavor, by means of herbs and spices, to sodium reduced product might be a viable solution [67]. Monosodium glutamate, which is responsible for the umami flavor, has been suggested as good flavor enhancer in low NaCl products, without substantially increasing the total sodium content of the product [59]. Kremer and colleagues replaced NaCl in a variety of foods, with different levels of naturally brewed soy sauce and measured the total NaCl and food acceptance [68]. They suggested that salad dressings (50% NaCl reduced), stir-fried pork (17% NaCl reduced) and soups (29% NaCl reduced), were still acceptable when NaCl was substantially decreased and substituted with soy sauce [68]. The study by Manabe [69] suggested that 17% of NaCl in egg custard could be decreased by the addition of dried bonito (fish) [69]. 

## 8. Concerns for Food Industry for Sodium Reduction

Sodium chloride is a widely used food additive due to its low cost, its ability to increase liking of foods via flavor modification, and other functional abilities in a food matrix [24,57]. Sodium chloride is required in food processing for some technological functions such as dough development in bread, and water binding and preservation in meats. However it is also believed that the sodium content of many food products exceeds technological requirements and primarily acts to enhance sensory effects [70]. A survey of UK food manufacturers revealed the most commonly cited function of sodium chloride in a range of food products was “to impart flavor” and the most commonly cited constraint to sodium reduction was “palatability and consumer preference” [71]. Given that sodium reduction will likely decrease preference for foods, there will be considerable pressure from food manufacturers to maintain current sodium levels in processed foods.

### 8.1. Loss of Palatability and Consumer Acceptance

Taste is one of the most important factors in food choice. Humans and animals have a liking for salt taste [27]. Therefore, when a large reduction in sodium content occurs there is a decline in consumer acceptance [72,73]. Small incremental decreases in sodium have shown to be effective, as consumers may not be able to detect gradual reductions in sodium up to a point [60]. However, with a continual decline of sodium from products it is inevitable that a point will be reached where a difference in flavor profile will be detectable by consumers and the food will be less liked. A recent study by Lucas *et al.* [54] showed that a significant sodium reduction (*i.e.*, >50%) of a meal component (hash brown) could occur with only a minor decrease in liking [54]. Therefore, large sodium reductions in foods are possible, but ideally the salt taste elicited by sodium needs to be replaced so that the consumer acceptance is not negatively affected [1].

### 8.2. Texture and Other Quality Characteristics

Reducing sodium chloride level may affect texture and other quality characteristics including, moisture levels, fat content, pH, starter cultures, various additives, and processing conditions [1]. For example, sodium chloride is able to bind proteins and fats and hold water. Therefore, meat batters with low sodium need to have a sodium replacer, which not only replaces salt taste, but also needs to compensate for the other functions, which are lost when sodium is decreased [74].

Sodium chloride also limits the growth of yeast and enables the gluten structure in bread to develop. The reduction of sodium chloride in bread may therefore result in an increase in the growth of yeast and an undeveloped gluten structure, which has a negative effect on the texture of bread [75]. Furthermore, reduction of sodium chloride in cheese may affect starter culture activity. This negative effect of sodium reduction, however, did not prevent food industry in the U.S. to market Cheddar cheeses with different levels of sodium chloride [76].

### 8.3. Preservation and Microbial Safety

Sodium reduces water activity in foods and is therefore able to limit the growth of pathogens and spoilage organism in a variety of food system [67]. For example, in processed meats and cheeses sodium chloride limits the growth and production of toxin by Clostridium Botulinum [77]. Similarly, sodium diacetate and sodium lactate limit the growth of *Listeria monocytogenes* and lactic acid bacteria in ready-to eat meats, which makes it safe for human consumption [78]. Sodium reduction may therefore pose a risk for unwanted bacteria growth and shorten shelf life. When sodium is reduced extra care needs to be taken in terms of cooking, packaging and storage temperatures. Furthermore, other preservatives may need to be added and all newly developed low sodium products need to be tested on microbiological safety and shelf life [67].

### 8.4. Other Functions of Sodium

In addition to safety risks and processing challenges involved in producing low sodium foods, there is also an economic consideration. Sodium chloride is relatively cheap and any substitute used will increase the cost of the product. Furthermore, in order to find suitable salt replacers, bitter blockers and or flavor enhancers substantial effort and money needs to go into research, development and consumer testing. The food industry will need to take all functions of sodium in foods into account when addressing sodium reduction.

## 9. Conclusion

Saltiness is an important sensory attribute of many foods, and sodium chloride contributes more than just saltiness to the characteristic flavor of many food types. Whilst ensuring adequate dietary sodium intake is vital to health, our intake of excessive amounts of sodium has been linked to development of hypertension and subsequent pathologies [79]. Changing the dietary sodium content of a population that has adapted to a high sodium diet will not be easy, and will entail a number of strategies [24]. Part of the reason previous attempts to reduce sodium in processed foods were not successful was due to loss of palatability of foods [73]. One strategy to reduce sodium is to replace sodium with potassium salts, and while potassium chloride elicits weak saltiness, at higher concentrations it also elicits metallic and bitter taste limiting its utility in food. The “stealth” approach of gradual sodium reduction in processed foods [60], thereby modifying consumers’ salt taste experience over time is recognized as arguably the best current strategy to reduce sodium in foods. Initiatives carried out in several countries will provide much-needed data about whether this approach is successful in reducing sodium intake and blood pressure at the population level. 

There are potential barriers such as the technological function of sodium in foods, and decreased consumer liking, that could be put forward by the food industry to stop sodium reduction. However, mandating sodium reduction in certain food categories would urge food industry to come up with creative solutions, which will enable sodium reduction without compromising on food safety and consumer acceptance. There is no doubt that sodium reduction in foods will be difficult, but equally there is no doubt that reducing the level of sodium in foods is essential for population health.
